# Revision of Surgery for Adolescent Idiopathic Scoliosis: Reasons, Treatments, and Clinical Management with Case Examples

**DOI:** 10.3390/jcm13082233

**Published:** 2024-04-12

**Authors:** Lawrence G. Lenke, Veronica Lee, Fthimnir M. Hassan

**Affiliations:** 1 Department of Orthopaedic Surgery, Columbia University Irving Medical Center, New York, NY 10032, USA; ll2989@cumc.columbia.edu; 2 The Daniel and Jane Och Spine Hospital, New York Presbyterian, Columbia University Irving Medical Center, New York, NY 10034, USA; 3 Roy and Diana Vagelos College of Physicians and Surgeons, Columbia University, New York, NY 10032, USA; vml2138@cumc.columbia.edu

**Keywords:** adolescent idiopathic scoliosis, revision, complications

## Abstract

Adolescent idiopathic scoliosis (AIS) is a curvature of the spine that develops in children ages 10–18 and can be attributed to unknown causes. The Lenke AIS classification system provides a template to classify these deformities by curve type paired with recommended operative treatments. Treatment of this patient population has been associated with low complication rates and overall surgical success. Nonetheless, a fraction of patients remain susceptible to revision surgery. This manuscript will focus on the aspects of AIS surgery, highlighting case examples, the different treatment approaches, complication rates, and primary reasons for revision surgery and associated outcomes.

## 1. Introduction

Adolescent idiopathic scoliosis (AIS) is a curvature of the spine that develops in children ages 10–18 and cannot be attributed to other causes of scoliosis, such as neuromuscular disorders or vertebral malformations [1]. AIS affects 1–3% of children and often occurs around the age of puberty, which can range between the ages of 11 and 18 years old [1]. Rates of AIS are higher in females than males, with female-to-male ratios ranging from 1.5:1 to 3:1, and this difference becomes more pronounced with increasing curve magnitude [2].

Introduced in 2001, the Lenke classification system of AIS provides an accurate and reliable system to classify idiopathic spinal deformities and recommended operative treatment approaches [3]. This system distinguishes six curve types (1–6), three lumbar spine modifiers (A, B, C), and three sagittal thoracic modifiers (−, N, +) [3]. The six curve types evaluate the type of curvature (major, minor structural, or nonstructural) in each of the major spinal column regions: proximal thoracic (PT), main thoracic (MT), and thoracolumbar/lumbar (TL/L) [3]. The lumbar modifier compares the position of the center sacral vertical line (CSVL) to the apex of the lumbar curve [3]. Lastly, the sagittal thoracic modifiers evaluate the T5-T12 sagittal Cobb measurement [3].

Importantly, the Lenke AIS classification system can indicate an operative treatment plan for AIS patients with Cobb angles over 40–50 degrees. One standard treatment for AIS is complete fusion of both the major and minor structural curves. Although complete fusion may decrease the potential risk of some post-operative complications, such as curve progression, it significantly limits the mobility of the spine and increases the risk of sagittal decompensation, lumbar degeneration, and chronic back pain [4]. In recent years, surgery for AIS has aimed to both prophylactically prevent curve progression and preserve mobility and flexibility among this young patient population. As such, selective fusion of the thoracic and thoracolumbar/lumbar curves has been increasingly used [5]. In these cases, selective fusion of the major curve leaves the minor curve unfused, allowing for spontaneous correction of the minor curve postoperatively.

Selective thoracic fusion of 1C and 2C curves (and some 3C curves) have shown to be successful, whereas 5C or 6C curves can be significantly improved with selective thoracolumbar/lumbar fusion [5]. With the proper instrumentation and level selection, spontaneous correction of the minor curve can be observed [5,6,7,8]. Vertebral body tethering (VBT), however, is another surgical procedure for AIS patients which seeks to preserve mobility and allow for spinal growth by linking vertebral bodies together with a flexible tether. Studies have reported VBT to be effective and safe, especially in young patients that have moderate curvature of the spine [9].

## 2. Case Discussions

**Figure 1:**
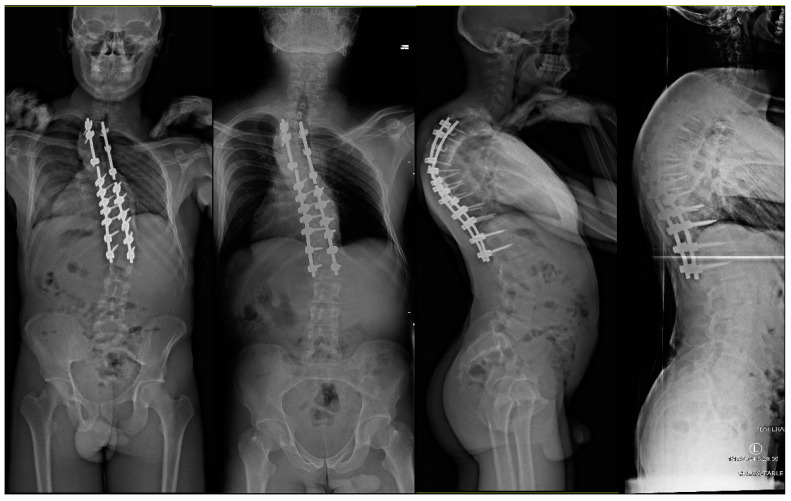
25-year-old male who previously underwent a posterior spinal instrumented fusion (PSIF) for T2-L1 at the age of 17 years presented with progressive back pain in the setting of bilateral rod fractures from T6 to T8. Preoperative anterio-posterior (AP) standing full-body radiographs demonstrated an 82° proximal thoracic and 65° main thoracic curve. Supine images showed the proximal and main thoracic curves decreasing to 77° and 48°, respectively. Preoperative lateral standing full-body radiographs demonstrated 88° of thoracic hyperkyphosis, 78° of hyperlordosis, and a small pelvic incidence of 48°.

**Figure 2:**
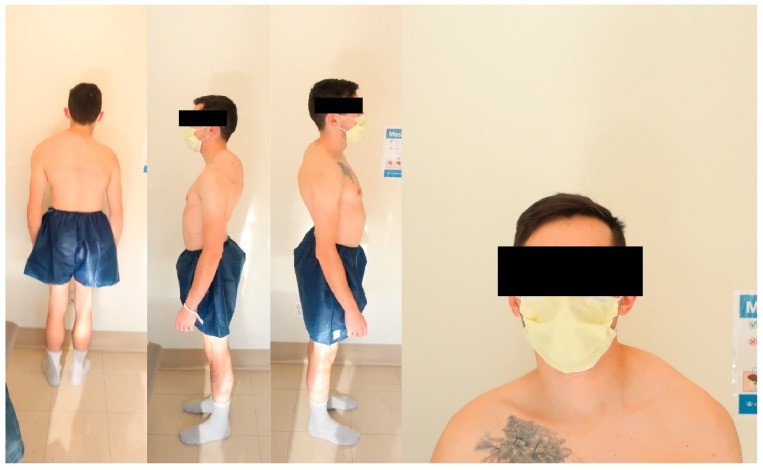
A 25-year-old male who previously underwent a posterior spinal instrumented fusion (PSIF) for T2-L1 at the age of 17 years presented with progressive back pain in addition to shoulder malignment and a large rib prominence secondary to his progressive deformity. The patient had a normal dynamic and static motor exam in his lower extremities with no evidence of any myelopathy.

Figure 1 and Figure 2 demonstrate a 25-year-old male who was diagnosed with AIS at the age of 14 years. After conservative treatments for three years, the patient underwent a posterior spinal instrumented fusion (PSIF) for T2-L1 at the age of 17 years. Nine years following the initial surgery, the patient presented with progressive back pain in the setting of bilateral rod fractures from T6 to T8 in addition to shoulder malignment and a large rib prominence secondary to his progressive deformity (Figure 2). The patient had a normal dynamic and static motor exam in his lower extremities with no evidence of any myelopathy. Preoperative anterio-posterior (AP) standing full-body radiographs (EOS Imaging, Paris, France) demonstrated an 82° and 65° proximal thoracic and main thoracic curve, respectively. Supine images showed the proximal and main thoracic curves decreasing to 77° and 48°, respectively. Preoperative lateral standing full-body radiographs demonstrated 88° of thoracic hyperkyphosis, 78° of hyperlordosis, and a small pelvic incidence of 48° (Figure 1).

**Figure 3:**
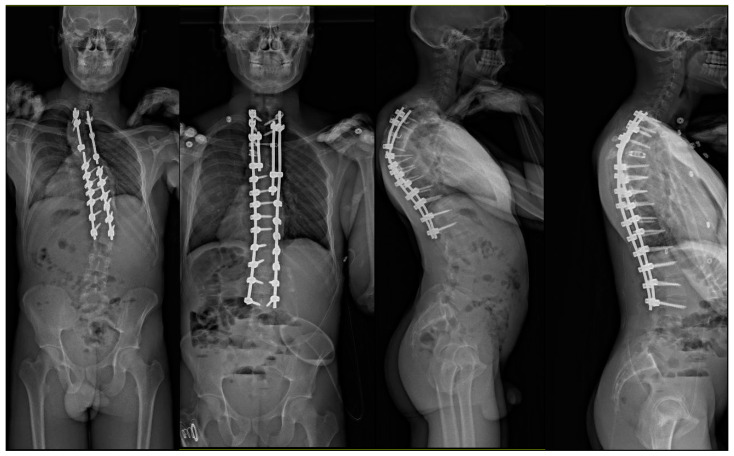
25-year-old male who underwent a posterior spinal instrumented fusion (PSIF) for T2-L1 at the age of 17 years presenting with progressive back pain in setting of bilateral rod fractures from T6 to T8. Patient underwent a revision surgery consisting of removal of the existing instrumentation from T3 to L1, PCOs from T2 to T12, VCR at T4 and T5, ASF at T3–T5 with the addition of an anterior structural titanium cage in the VCR site, and PSIF from T1 to L3. The procedure went without uncomplications with excellent correction obtained.

**Figure 4:**
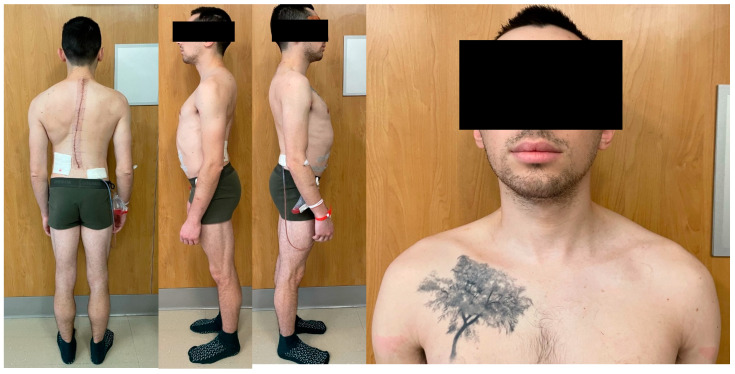
A 25-year-old male who underwent a posterior spinal instrumented fusion (PSIF) for T2-L1 at the age of 17 years who presented with a progressive deformity clinically highlighted by shoulder malignment and a large rib prominence. Following his revision surgery consisting of ROI T3-L1, PCO T2–T12, VCR T4–T5, ASF T3–T5, PSIF T1-L3, excellent correction of his deformity was obtained both radiographically and clinically.

The patient underwent a revision surgery consisting of removal of the existing instrumentation from T3 to L1, posterior column Smith-Petersen osteotomies from T2 to T12, vertebral column resection (VCR) via a lateral extracavitary approach at T4 and T5, anterior spinal fusion for T3–T5 with the addition of an anterior structural titanium cage in the VCR site, and PSIF from T1 to L3. The procedure went uncomplicated with excellent correction obtained both radiographically and clinically (Figure 3 and Figure 4).

## 3. Discussions

### 3.1. Complications and Reasons for Revision

Perioperative complication rates associated with surgical treatment of AIS have been reported to be between 5% and 23% [10,11,12,13]. More specifically, a study by Menger et al. [14] estimated rates of 0.9% for neurological complications, 2.8% for respiratory complications, 0.8% for cardiac complications, 0.4% for infections, 2.7% for gastrointestinal complications, 0.1% for renal complications, and 0.1% for venous complications. Following hospital discharge, postoperative complications can occur at a rate of around 4.1%, with the majority being wound- and instrumentation-related complications. Early postoperative complications include instrumentation malposition, poor wound healing/wound dehiscence, and postoperative infection [11]. Regarding instrumentation malposition, malpositioned screws and loss of fixation to bone or connection between implants make up the majority of cases [11]. Regarding postoperative infection, deep infection occurred in 31.4% (11 of 35 cases), while superficial infection occurred in 25.7% (9 of 35 cases) [11]. The most common longer term postoperative complications are pseudarthrosis, curve progression, and proximal junctional kyphosis [15]. Among these, postoperative infection, pseudarthrosis, and curve progression are the most frequent causes of revision surgery, accounting for 34%, 26%, and 17% of reoperations, respectively [16]. In an analysis of 36,335 patients, De la Garza Ramos et al. [17] found rates of postoperative complications to be higher among patients who are male, younger, and have anemia, hypertension, or hypothyroidism.

A study by Jamnik et al. [18] found a greater than 50% decrease in overall complication rate of patients treated in 2013–2019 compared to those treated in 2008–2012, suggesting that surgery for AIS is continuing to improve in treatment efficacy and patient safety. Interestingly, this decrease was due to decreases in rates of postoperative infection and symptomatic instrumentation, rather than the rate of complications due to pseudarthrosis and instrumentation failures, which have remained unchanged [18].

Regarding selective fusion surgeries specifically, studies have shown this technique to have excellent long-term results with low rates of postoperative complications [19,20]. For example, in a retrospective study that followed AIS patients after undergoing selective thoracic fusion with a minimum 5-year follow-up, Suk et al. [21] reported no junctional kyphosis, coronal decompensation in 4.9% of patients (10 of 203 patients), and curve progression in 8.4% of patients (17 of 203 patients). Another study by Edwards et al. [8] followed 44 consecutive AIS patients who underwent selective thoracic fusion for 2–16 years after corrective surgery and found that none of the patients required postoperative bracing or revision operations. Furthermore, Edwards et al. [8] reported no instances of instrumentation loosening, dislodging, or breaking. Although some cases that utilized the posterior approach reported proximal junctional kyphosis at the thoracolumbar junction (preoperative to latest +5°), this was not noted for any cases that utilized the anterior approach [8].

Revision surgeries vary widely depending on the postoperative complications they aim to correct. For example, extension of instrumentation proximally or distally can address curve progression [22,23,24]. Placement of additional instrumentation can be added posteriorly or anteriorly to address pseudarthrosis and stabilize the spine [22,23,24]. In some cases, part or all of the instrumentation can be removed to address pain or infection [22,23,24]. Yet another revision technique employs osteotomies to address progressive spine deformity [22].

For selective thoracic fusions in particular, some studies report that improper choice of the lowest instrumented vertebra (LIV) leads to postoperative complications, most notably curve progression, and the need for revision [25,26]. In 18 such cases, El Rachkidi et al. [27] successfully employed the simple technique of extending the instrumentation and fusion to the LIV + 1 level. In these cases, all patients were female with a mean age of 13.9 years, and the mean time between the primary surgery and revision surgery was 8.4 months [27].

Revisions due to decompensation, adding on, and junctional kyphosis occur mainly 2–5 years after the primary operation, while revisions due to wound and instrumentation-related complications, such as infection and malposition, mainly occur less than 2 years after operation [15]. A study by El Rachkidi et al. [27] reported that patients were never re-operated on before 3 months postoperative to allow for spontaneous correction. Yet another study by Diederich et al. [28] suggests a bimodal timeline for revision surgery, with revisions occurring either within 15 years or over 30 years after the primary operation.

Regarding vertebral body tethering, the most common postoperative complications are tether breakage, pulmonary complications, and overcorrection [29]. Although successful cases are able to preserve spinal mobility, several studies point to relatively high rates of complication and revision surgery compared to fusion and selective fusion. For example, Newton et al. [30] found that 8 of the 17 patients in their study (47%) had a suspected broken tether, and 7 of the 17 patients (41%) underwent revision surgery to remove, add, or replace tethers. In another study, Zhang et al. [29] reported tether breakage in 21.3% of patients and a revision rate of 13.1%. For these reasons, selective fusion appears to be the safer option AIS patients.

### 3.2. Revision Surgery and Patient Reported Outcomes

Most AIS cases treated surgically are successful, and patients report substantial improvements in self-image and satisfaction, as well as moderate improvements in pain, functionality, and mental health at 2 and 5 years following the operation [31]. Although Mariconda et al. [32,33] suggests that these improvements did not lead to full normalization of health-related quality of life compared to age and sex-matched healthy controls 5 years after surgery, a study by Simony et al. [32,33] found that the health-related quality of life for AIS patients treated with spinal fusion was similar to that of the general population by the 25 year mark.

Despite the general success of spinal fusion for AIS patients, major postoperative complications have been reported to diminish patient-reported outcomes (PROs). Patients experiencing active major complications scored the lowest in all domains of the Scoliosis Research Society (SRS-22) questionnaire [34]. In addition, a study by Jamnik et al. [35] found that patients who experienced curve progression, 20% of whom underwent a revision surgery, had a significantly lower SRS score for appearance, satisfaction, and mental health 5 years after surgery.

## 4. Conclusions and Trajectory Moving Forward

Surgery for AIS patients will continue to show lessened complications with improved outcomes as both fusion and tether surgery become more standardized. In the future, machine learning algorithms will undoubtedly assist surgeons and parents in determining the best surgical approach at the optimal time for the most successful result with the lowest complication rates.

## Data Availability

No data were reported as part of this study.
